# MaxI-Net: A 3D AI Framework for CBCT-Based Maxillofacial Defect Reconstruction and Patient-Specific Implant Generation with Biomechanical Validation

**DOI:** 10.3390/bioengineering13060619

**Published:** 2026-05-26

**Authors:** Mamta Juneja, Maanya Kharbanda, Nitin Pandey, Agrima Sudhir, Aditya Poddar, Harleen Kaur, Prashant Prakash, Manoj Kumar Jaiswal, Prashant Jindal, Philip Breedon

**Affiliations:** 1 University Institute of Engineering and Technology, Panjab University, Chandigarh 160014, India; mamtajuneja@pu.ac.in (M.J.);; 2 Oral Health Science Centre, Post Graduate Institute of Medical Education and Research, Chandigarh 160012, India; jaiswal.manojk@pgimer.edu.in; 3 Department of Engineering, School of Science and Technology, Nottingham Trent University, Nottingham NG1 4F, UK; 4 Medical Technologies Innovation Facility, School of Science and Technology, Nottingham Trent University, Nottingham NG1 4F, UK

**Keywords:** artificial intelligence, deep learning, medical image analysis, maxillofacial defect reconstruction, patient-specific implants, CBCT, finite element analysis, biomechanical validation, PEEK implants, 3D reconstruction

## Abstract

Maxillofacial defects impair facial aesthetics and oral function, arising from trauma, tumor resection, or congenital anomalies; however, reconstruction using Computer-Aided Design (CAD) and autologous grafts remains complex and time-intensive, and is associated with donor-site morbidity. Although deep learning (DL) has advanced automated reconstruction, existing models often address isolated tasks, lack integrated multi-scale feature learning, and rely on small datasets. This study proposes the Maxillofacial Implant-generation Network (MaxI-Net), a fast, resource-efficient three-dimensional DL framework for end-to-end maxillofacial defect reconstruction and patient-specific implant generation, with a completion step of cavity filling within the assembly. The model employs a 3D encoder–bottleneck-decoder architecture integrating hybrid dilated convolutions, residual connections, squeeze-and-excitation (SE) blocks, and 3D Convolutional Block Attention Modules (CBAM) with multi-scale feature fusion. It was trained on 921 Cone Beam-Computed Tomography (CBCT) scans, augmented to 11,973 maxillary defect pairs, using Dice loss and Adam optimisation with Automatic Mixed Precision, and benchmarked against UNet, UNETR, SegResNet, and SwinUNETR. MaxI-Net achieved the following: superior Dice Similarity Coefficient (DSC) = 0.778; 95th percentile Hausdorff Distance (HD95) = 3.453 mm; DSC Standard Deviation (SD) = 0.094; 95% confidence interval (CI) for mean DSC: 0.775–0.782). It was statistically validated against all competing architectures via pairwise Wilcoxon signed-rank tests, with significant DSC improvements confirmed across all comparators (*p* < 0.001) and rank-biserial effect sizes ranging from r = 0.250 against the closest competitor SegResNet* with high efficiency (0.06 s/volume; 9.6 min/epoch). Internal cavity filling of the generated implants was performed as a brief manual post-processing step in Autodesk Fusion 360 prior to biomechanical validation. Biomechanical validation using a finite element analysis (FEA) of polyether–ether–ketone (PEEK) implants (~26.53 g) showed 41% stress reduction under physiological loads (100–400 N), predicting a ~9.2-year lifespan.

## 1. Introduction

Maxillofacial reconstruction is a major challenge in head and neck surgery due to the intricate midface anatomy and its essential roles in facial aesthetics, mastication, and speech. Maxillary defects, caused by trauma, oncologic resections, osteoradionecrosis (ORN), or congenital deformities, significantly affect functional, psychological, and social well-being. Traditional reconstruction methods, including prosthetic rehabilitation and autologous bone grafting, are limited in terms of precision, patient morbidity, and increased surgical complexity [1]. Consequently, alloplastics, 3D-printed implants, and scaffolds have gained prominence [2].

Advances in medical imaging, CAD, additive manufacturing, artificial intelligence (AI), and DL have transformed craniofacial reconstruction by enabling automated segmentation, defect modelling, and implant generation, expanding reconstruction possibilities beyond manual workflows [3,4,5]. Notably, AI has been applied to maxillary structure segmentation, a task previously limited to the mandible [6], and can be leveraged for end-to-end defect reconstruction and patient-specific implant synthesis.

Early clinical approaches relied on obturator prostheses, providing rapid rehabilitation, but had limited long-term stability, especially for speech and swallowing [7]. CAD/Computer-Aided Manufacturing (CAM) technologies further enabled the precise fabrication of patient-specific implants using titanium, PEEK, Poly-DL-Lactic Acid (PDLLA), and calcium phosphate [8]. Material-focused studies, such as Polycaprolactone (PCL) scaffolds via 3D printing, demonstrated anatomical matching but limited osteoinductive potential [2]. The clinical feasibility of PEEK and titanium implants was confirmed in small cohorts with favourable short-term outcomes [9].

Kong et al. [10] proposed an Enhanced Encoder–Decoder Network (EED-Net) for panoramic X-ray segmentation, achieving high accuracy, while Wang et al. [11] showed that Virtual Surgical Planning (VSP) combined with 3D printing outperforms freehand surgery. The long-term reliability of titanium implants was validated by Lim et al. [12]. CBCT segmentation frameworks such as U-Net [13] improved diagnostic efficiency, and clinical integration using VSP and additive manufacturing demonstrated early, precise reconstruction in trauma, oncology, and Medication-Related Osteonecrosis of the Jaw (MRONJ) cases [14]. Anatomy-specific automation extended to zygomatic reconstruction with VGG16 + 3D U-Net [15], and panoramic-to-CBCT 3D reconstruction using Generative Adversarial Network (GAN)-based models reduced radiation exposure [16]. Melerowitz et al. [6] achieved detailed maxilla-mandible segmentation into 12 substructures, while Matros et al. [17] integrated VSP, 3D-printed guides, and immediate dental implants for oncologic reconstruction. Finally, multimodal Computed Tomography (CT)/Magnetic Resonance Imaging (MRI) fusion with deep learning enabled tumor-aware planning [18]. Table 1 summarises these studies. Table 1 provides insights into all these approaches.

In summary, prior work has progressively advanced maxillofacial reconstruction from prosthetic rehabilitation [7] to CAD/CAM implants [8,9,12], AI-driven segmentation and anatomical preprocessing [6,10,13,15,16], and integrated digital workflows [11,14,17,18]. However, most approaches remain limited by isolated workflows and small datasets, and rarely incorporate biomechanical validation, restricting scalability and clinical feasibility. To address these gaps, this study introduces the Maxillofacial Implant-generation Network (MaxI-Net), an end-to-end deep learning framework that is aimed to directly generate implant-ready maxillary and zygomatic reconstructions from CBCT data and validate the mechanical strength of these implants under human mastication forces, in an attempt to save time and resources and scale up the implant design and reconstruction process.

Key Novel Contributions of MaxI-Net:

1. **Direct Implant-Ready AI Pipeline for Maxillary-Zygomatic Reconstruction:** Generates patient-specific, ready-to-manufacture implant geometries directly from CBCT volumes, substantially reducing manual CAD-based design steps, with a brief internal cavity-filling reinforcement step as post-processing.
2. **Clinically Guided Synthetic Defect Simulation Strategy:** Controlled defect injection, adapted from Brown’s oncologic classification, producing 11,973 paired healthy-defected volumetric samples for robust supervised learning.
3. **Joint Modelling of Maxillary and Zygomatic Structures:** Utilises simultaneous reconstruction of both regions within a single framework, capturing midfacial interdependencies encountered in trauma and oncologic cases.
4. **Large, Expert-Curated Volumetric Dataset:** Utilises 921 clinically acquired CBCT scans, expanded to 11,973 pairs across seven anatomical regions, enabling learning of consistent anatomical priors.
5. **Hybrid Multi-Scale Attention-Driven 3D Architecture:** An advanced encoder–decoder design integrating residual learning, hybrid dilated convolutions, and 3D attention mechanisms, achieving high reconstruction accuracy (DSC = 0.778; HD95 = 3.453 mm), was statistically validated against all competing architectures via pairwise Wilcoxon signed-rank tests (*p* < 0.001) with rank-biserial effect sizes up to r = 0.971, with efficient Adam optimisation with Automatic Mixed Precision.
6. **Biomechanically Validated Lightweight Implant Design:** Finite Element Analysis of AI-generated PEEK implants demonstrates a 41% reduction in peak von Mises stress under physiological loading (100–400 N), enabling lightweight (~26.53 g), long-lifespan (~9.2 years) implant designs.
7. **Resource-Efficient 3D Inference:** Ultra-fast volumetric reconstruction (0.06 s per volume) and efficient training (9.6 min per epoch) enable scalable deployment, particularly in resource-constrained clinical settings.
8. **Clinically Verified Implant Fit and Anatomical Accuracy:** Surgeon-evaluated implants show accurate anatomical alignment, edge continuity, and functional suitability for real-world maxillofacial reconstruction.

## 2. Materials and Methods

**Figure 1:**
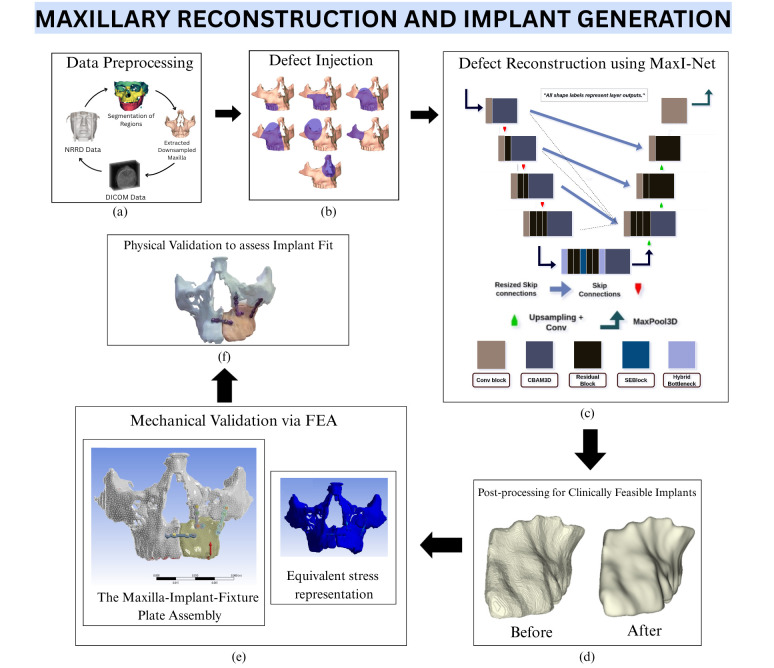
Proposed Framework for Maxillofacial Defect Reconstruction: (**a**) Data Preprocessing, (**b**) Defect Injection, (**c**) Defect Reconstruction using MaxI-Net, (**d**) Post-processing for Clinically Feasible Implants, (**e**) Mechanical validation via FEA, and (**f**) Physical Validation to assess Implant Fit.

The proposed framework for maxillofacial defect reconstruction consists of three primary stages, as illustrated in Figure 1. The first stage involves the preparation of the dataset for training the deep learning model, including (a) data preprocessing and (b) defect injection for data augmentation (Figure 1a,b). The second stage comprises the design and implementation of MaxI-Net, a novel deep learning model for defect reconstruction (Figure 1c). The third stage involves the post-processing of the reconstructed outputs to generate clinically feasible implants (Figure 1d), which includes a brief manual internal cavity filling step in Autodesk Fusion 360 for structural reinforcement prior to evaluation. This is followed by mechanical validation to assess biomechanical performance (Figure 1e) and physical validation to evaluate implant fit (Figure 1f).

### 2.1. Dataset Preparation

The data preparation stage consists of several steps, such as data acquisition, the conversion of file formats, segmentation using the Automatic Multi-Anatomical Skull Structure Segmentation (AMASSS) Module, resampling, and defect injection. The sections that follow elaborate upon each of these steps.

#### 2.1.1. Dataset Acquisition

Clinical data were obtained from the Oral Health Sciences Centre (OHSC), Post Graduate Institute of Medical Education and Research (PGIMER), Chandigarh, India. The dataset comprised CBCT scans in a Digital Imaging and Communications in Medicine (DICOM) format. A total of 1490 scans covering the lower and midfacial regions, including the mandible, maxilla, and zygomatic bones, were included, while upper facial and cranial regions were excluded. Ethical approval was obtained from the Institutional Ethics Committee of PGIMER, Chandigarh (IEC No.: IEC-01/2025-3317; Ref No.: PGI/IEC/2025/EIC000469), and all data were anonymised prior to analysis, with informed consent obtained or waived as per institutional guidelines.

#### 2.1.2. Data Conversion

The DICOM files presented several limitations for direct use in further processing. These included the presence of non-essential anatomical structures such as the mandible, skin, and soft tissues. For removal of the above-mentioned unwanted structures, the file format was converted from DICOM to Nearly Raster Raw Data (NRRD), which is a more flexible format and can be used with different software programs. It enables more efficient analysis and alteration of the medical images, which facilitates their usage in subsequent procedures.

#### 2.1.3. Maxilla Segmentation

The next step in the preprocessing pipeline involved the segmentation of the maxillary bone from surrounding anatomical structures. This was accomplished using the AMASSS–CBCT module from the Slicer Automated Dental Tools extension within 3D Slicer [19]. The segmentation accuracy was configured at 80% to adhere to time and memory constraints, which yielded high-quality maxillary bone segmentations suitable for further analysis. This initial dataset of segmented maxillae included many non-viable files, such as those showing defects, post-surgical plates, or inaccuracies from the segmentation process. To ensure data quality, all files were visually inspected and segregated. This curation process yielded a final, clean dataset consisting of 921 healthy files.

#### 2.1.4. Downsampling

To reduce computational overhead, all volumetric data were downsampled using nearest neighbour interpolation, preserving anatomical structure while optimising memory and processing efficiency.

#### 2.1.5. Defect Generation and Augmentation

Defect generation, following segmentation and downsampling, was a crucial step for dataset augmentation to train the reconstructive model. The process involved identifying appropriate anatomical regions for defect creation, followed by injecting the defects into those regions. The defects spanned seven anatomical regions, classified into six types of vertical maxillofacial defects and one zygomatic defect, adapted from the existing clinical literature [20,21] but simplified for computational modelling. Each defect type is denoted using an acronym (in brackets), which will be used consistently throughout this paper as given in Table 2.

**Figure 2:**
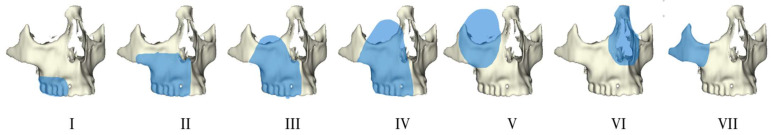
Regions for Defect Injection (Classes I–VII).

The above regions are illustrated in Figure 2.

**Figure 3:**
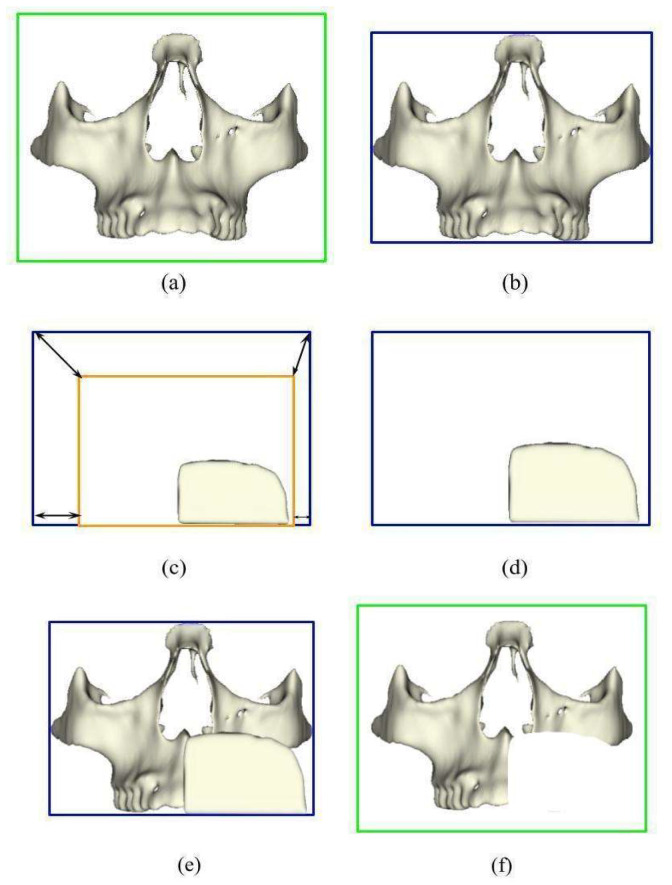
Schematic representation of the Defect Generation Methodology. (**a**) Original Maxilla NRRD with Dimensions X × Y × Z (Green), (**b**) Maxilla NRRD Cropped to Bounding Box with dimensions X’ × Y’ × Z’ (Blue), (**c**) Original Mask with Dimensions X″ × Y″ × Z″ (Orange), (**d**) Interpolated Mask with dimensions X’ × Y’ × Z’, (**e**) Application of Defect Mask through Binary Subtraction and (**f**) DefectedMaxilla File after Padding to Retain Original dimensions X × Y × Z.

The methodology utilised for generating defects was carefully designed to prevent any changes to the data of the NRRD file, except for generating the desired defect. Therefore, the dimensions of the file before and after defect generation remained constant. Figure 3 shows the steps involved in the defect generation process, with file dimensions abstractly portrayed as 2D rectangles around an NRRD file. Each coloured rectangle represents a different set of dimensions. In the figure, the input maxilla NRRD file is shown to have dimensions X × Y × Z (represented in green color in Figure 3a), where X, Y, and Z are numbers denoting voxel counts along the three principal axes. The file, when cropped around its bounding box, results in dimensions X’ × Y’ × Z’ (represented in blue in Figure 3b). The original dimensions of the mask are assumed to be X″ × Y″ × Z″ (shown in orange in Figure 3c).

The approach utilised the creation of defect masks for each defect type using 3DSlicer (5.8.0 Version) [19], which would be applied to each healthy file for creating defects. The application of a mask is synonymous with the binary subtraction of the mask from the healthy file, resulting in a defected file. To address the issue of mismatching dimensions of the maxilla NRRD file and the mask, the mask was interpolated to match the dimensions before the subtraction process, as shown in Figure 3c,d. A critical step in the methodology to ensure the precise location of the injected defects was bounding box-based cropping of the maxilla files as an intermediate step, followed by defect injection through mask subtraction (Figure 3e) and padding to restore original dimensions (Figure 3f). The cropping step ensures that the relative position of the mask is accurate for each maxilla, thus creating defects in the desired region. A total of 13 defect masks were used for creating the defects in the identified regions, which contributed significantly to the augmentation process. The process was followed by the application of Connected Component Analysis (CCA) to the files to ensure the retention of only the maxillary zygomatic region of interest and the removal of any other unwanted structures in the NRRD file. Subsequently, the files were resampled again before being input into the model.

### 2.2. AI Modelling

The architectural choices and training strategies adopted to design and implement a deep learning-based model for maxillofacial reconstruction are provided in this section. The section starts with the details of layers and operations used in the encoder, followed by the explanation of the hybrid dilated convolutions, 3D CBAM Attention [22], Residual and SE blocks [23] utilised in the bottleneck, and, finally, the skip connections with multi-scale feature fusion and activation functions utilised in the decoder. The training strategy utilised for the model is then discussed.

#### 2.2.1. Model Architecture: MaxI-Net

**Figure 4:**
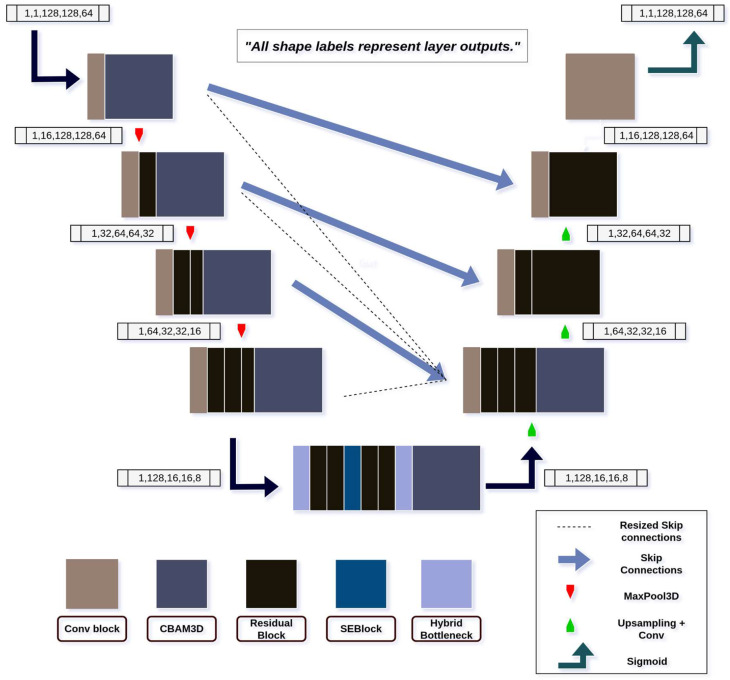
Proposed MaxI-Net Architecture.

Figure 4 shows a graphical representation of the proposed Maxillofacial Implant-generation Network, MaxI-Net. The model is a 3D convolutional neural network designed with a focus on increasing defect reconstruction quality. The encoder-bottleneck-decoder layout is enhanced with the inclusion of widely known state-of-the-art architectural components, bringing together the functionalities of each element to improve the model’s performance.

The encoder progressively extracts increasingly abstract features while retaining essential spatial dependencies. The four hierarchical stages consist of a convolutional block with stacked 3 × 3 × 3 kernels followed by group normalisation and the application of the Gaussian Error Linear Unit (GELU) [24] activation function. A common feature at each level is the utilisation of 3D CBAM, which applies both Channel Attention (CA) and Spatial Attention (SA) to highlight informative channels and spatial locations.

Subsequent stages incorporate residual blocks to improve gradient flow and feature reuse. The channel depth is increased two-fold at each stage, while 2 × 2 × 2 max-pooling performs downsampling between stages. A hybrid dilated convolution module, employing dilation rates of 2 and 4, is utilised to enlarge the receptive field, followed by Group Normalisation and GELU activation. A SE then applies global average pooling and channel-wise reweighting to emphasise relevant features. Multiple residual refinement blocks with dropout regularisation further enhance the feature representations. The bottleneck concludes with a hybrid dilated module and CBAM attention block for final feature enhancement before decoding. The decoder consists of the following operations: In the first decoding stage, the upsampled bottleneck output (having 128 channels) is concatenated with the Stage 3 encoder output (having 64 channels) and multi-scale features (adding 48 channels), resulting in a 240-channel tensor. This tensor passes through convolutional and residual blocks followed by the 3D CBAM block. Multi-scale feature fusion ensures consistent spatial context during decoding. In this, the encoder outputs from multiple stages are reduced to 16 channels via 1 × 1 × 1 convolutions and resampled with trilinear interpolation to match the required decoder resolutions. This enables the decoder to integrate spatial information from various hierarchical levels. Stages 2 and 3 repeat the upsample-and-concatenate process with their respective encoder features, each followed by convolutional refinements. The final layer applies a 1 × 1 × 1 convolution to project the features to the target output dimension, followed by a sigmoid activation for voxel-wise predictions.

Special features of the model include the use of attention mechanisms throughout the architecture. The 3D CBAM sequentially applies channel and spatial attention to adaptively recalibrate feature maps, while SE blocks in the bottleneck selectively enhance the most relevant channels. These modules strengthen the network’s ability to focus on critical anatomical structures while suppressing irrelevant background information. The network uses GELU and Sigmoid Linear Unit (SiLU) activations for smooth nonlinear transformations and employs normalisation layers (BatchNorm3D and GroupNorm) to stabilise training and mitigate internal covariate shift. The integration of hybrid dilated convolutions, residual and SE blocks, multi-scale feature fusion, and 3D CBAM attention into a unified framework allows the model to capture both fine anatomical details and large-scale spatial structures, making it highly effective for 3D reconstruction tasks.

#### 2.2.2. Training Strategy

The training specifications of MaxI-Net included using Dice loss as a loss function along with utilising Adam Optimiser [25]. Automatic Mixed Precision (AMP) was enabled to increase computational efficiency and minimise GPU memory usage. The Rich library was utilised for progress monitoring, while the learning rate was adaptively adjusted according to the validation loss using the ReduceLROnPlateau scheduler with a patience of five epochs and a decay factor of 0.5. Furthermore, cudnn.benchmark was set to True to further enhance training speed. The model was trained to produce defect reconstructions as the output, given an input defectied maxilla. The augmented dataset produced was divided into the train, validation, and test sets. Across all training sessions, the configurations such as loss function, optimiser, and batch were kept the same. Additionally, model weights were saved every 10 epochs, and the set of weights corresponding to the lowest relative validation loss was also stored.

#### 2.2.3. Time Efficiency Analysis

The state-of-the art models were compared with the proposed model on the basis of training time per epoch and inference time. 

### 2.3. Post-Processing

The initial 3D reconstructions generated by MaxI-Net were reviewed by a maxillofacial surgeon and required post-processing prior to physical and biomechanical validation. The post-processing workflow was performed in a structured sequence.

First, surface smoothing of the reconstructed defect was carried out using 3D Slicer. An appropriate smoothing factor was selected on a case-by-case basis, typically between 0.5 and 1, and the resulting model was exported in Standard Tessellation Language (STL) format.

Next, an alignment step was performed by virtually positioning the reconstructed implant within the defect cavity to ensure correct anatomical registration. Any intersecting regions between the maxilla and the reconstructed implant were then removed in Autodesk Fusion 360 [26] using Boolean subtraction. Small clearance gaps were additionally introduced between the defect boundary and the reconstructed implant to facilitate proper seating and surgical fit.

Finally, a brief manual reinforcement step was performed in Autodesk Fusion 360, where internal cavities within the AI-generated implant were filled using a boundary-fill operation. This step was introduced to improve the structural continuity of the implant prior to biomechanical evaluation and was completed in under two minutes per case. This reinforcement ensured adequate internal support for fixation components under physiological loading conditions.

These post-processing steps collectively ensure geometric accuracy, surgical fit, and biomechanical readiness of the final implant assembly for subsequent validation procedures.

### 2.4. Mechanical Testing

Once the implant design was finalised, its mechanical performance was evaluated under physiologically relevant mastication conditions. A complete Maxilla–Implant–Fixture plates assembly was designed using Finite Element Method (FEM), and its biomechanical behaviour was assessed through FEA. Two types of mechanical tests were conducted: a static load analysis and a fatigue analysis to simulate realistic functional loading. Details of each step are provided in the following subsections.

#### 2.4.1. Designing Complete Maxilla Implant Fixture Assembly

A complete maxilla implant fixture plate assembly was designed in Autodesk Fusion 360 to simulate human chewing forces. The assembly included the defectied maxilla, the AI-generated implant, and fixation components consisting of two Ti-6Al-4V (Titanium Alloy) linear four-hole plates and one L-shaped plate, secured with matching alloy pins. Linear plates provide better resistance against tensile and compressive loading, while L-shaped plates improve resistance against rotational forces and torque [27].

**Figure 5:**
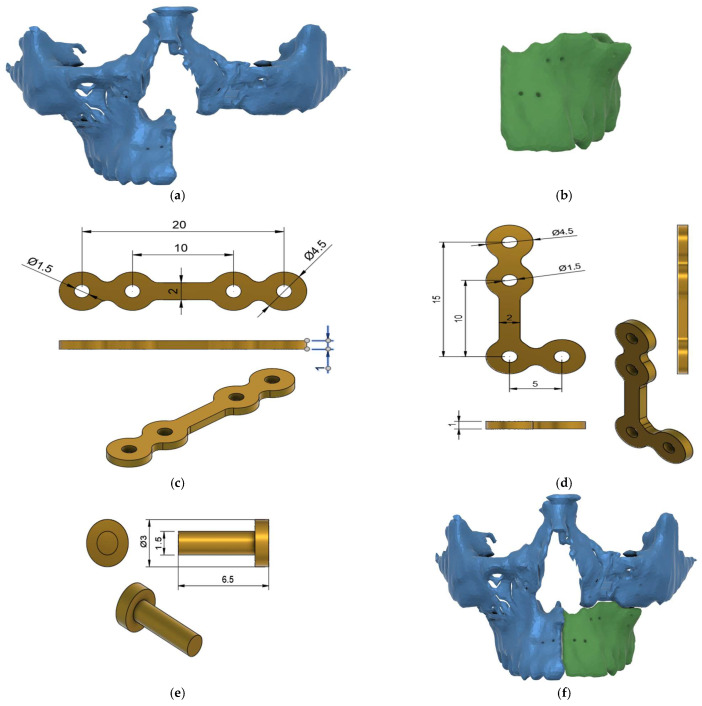

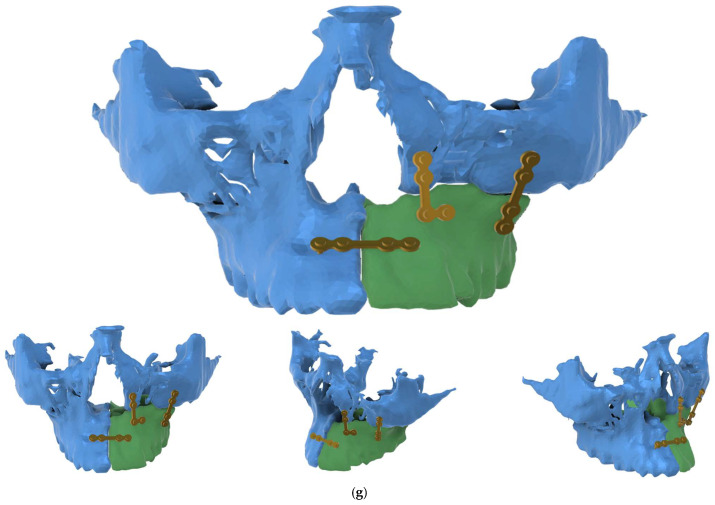
(**a**) Defectied Maxilla, (**b**) AI-generated Implant, (**c**) Linear four-hole Titanium Alloy Fixture Plate, (**d**) L-shaped Titanium Alloy Fixture Plate, (**e**) Titanium Alloy Fixture Pins, (**f**) Aligned Implant and Maxilla, and (**g**) Complete Assembly of Defectied Maxilla and AI-generated Implant Fixed with Linear and L-shaped Titanium Alloy Fixture Plates, Secured using Pins (All Dimensions in mm).

Fixture plate positioning followed recommendations from an experienced maxillofacial surgeon to ensure stringent support at the maxilla implant interface and to prevent slippage that could lead to cascading mechanical failure. The implant was precisely seated within the defect cavity such that a uniform boundary contact was maintained. Boolean subtraction was then applied to create placement holes for the fixture components in both the defectied maxilla and implant. Figure 5 illustrates the defectied maxilla (a), AI-generated implant (b), linear plate (c), L-shaped plate (d), titanium pins (e), aligned implant (f), and the final assembled structure (g).

#### 2.4.2. Load Testing Using FEA

The finalised maxilla–implant assembly was evaluated through static and fatigue analyses. Static analysis simulated a constant vertical force on the assembly to assess immediate structural response, while fatigue analysis applied cyclic, half-reversed loading at 1 Hz to replicate repetitive mastication [28]. Three force magnitudes were considered: 100 N (typical chewing) [29], 250 N (above-normal chewing), and 400 N (average maximum bite) [30]. This evaluation captured the structural behaviour of the maxilla–implant–fixture system under continuous masticatory loads, enabling the assessment of potential fatigue and overall biomechanical reliability.

### 2.5. Physical Validation to Assess Implant Fit

This section describes the physical validation protocol used for assessing the generated maxillofacial implants. A representative case from the test set was selected for fabrication. The defectied maxilla and corresponding post-processed implant were converted to STL format and 3D printed. The printed implant was assembled with fixation plates to replicate the surgical configuration. Geometrical fit assessment was performed by a maxillofacial surgeon from PGIMER, Chandigarh, focusing on alignment, edge contact, and surface continuity of the assembled model.

### 2.6. Assessment and Validation on a Patient Case

This section describes the clinical validation protocol using a real patient case obtained from OHSC, PGIMER, Chandigarh, which was not included in model training or testing. MaxI-Net was applied to reconstruct a large unilateral maxillary defect and generate a patient-specific implant. The reconstructed model was evaluated for anatomical alignment and spatial correspondence with the surrounding maxillary structures. The output was reviewed by a maxillofacial surgeon for the assessment of anatomical reconstruction quality and potential suitability for implant planning.

## 3. Results

The experiments were conducted on a high-performance computing platform equipped with an Intel Xeon W5-2465X CPU, featuring 16 physical cores and a maximum clock speed of 3.1 GHz. This system was paired with an Nvidia RTX A6000 GPU, which includes 48 GB of GDDR6 memory, 18,176 CUDA cores, and 568 Tensor Cores. Additionally, the GPU supports multiple high-resolution displays through DisplayPort 1.4 interfaces.

### 3.1. Dataset Preparation Results

**Figure 6:**
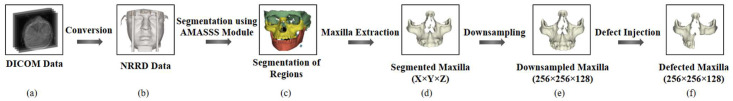
Results of Data Preparation Pipeline. (**a**) DICOM Data, (**b**) NRRD data, (**c**) Segmentation of Regions, (**d**) Segmented Maxilla, (**e**) Downsampled Maxilla, (**f**) Defectied Maxilla.

The preprocessing and augmentation methods explained in Section 2.1 were applied to the acquired dataset. Figure 6 shows the results of the complete data preparation pipeline in a sequential manner. The outcome of each step is discussed in this section.

#### 3.1.1. Data Acquisition

A total of 1490 CBCT scans were initially obtained from OHSC, PGIMER and subsequently converted from DICOM to NRRD format, as shown in Figure 6a,b. Following careful quality checks through visualisation, 921 of the 1490 NRRD files were found to be anatomically complete and suitable for further processing. These files were devoid of maxillofacial defects or segmentation errors.

#### 3.1.2. Maxilla Segmentation Results

Using the AMASSS–CBCT Slicer Module, folders containing unsegmented NRRD files were processed to obtain the segmented maxilla. The module first produced a segmentation of different regions, as shown in Figure 6c. Thereafter, all components except the maxilla were set to 0, whereas the maxillary and zygomatic regions were set to 1, resulting in a binary NRRD file, as shown in Figure 6d. The original dimensions of the file are assumed to be X × Y × Z, where X, Y, and Z denote the number of voxels along the three principal axes.

#### 3.1.3. Resampling

By using nearest neighbour interpolation technique, the NRRD files were downsampled from a resolution of X × Y × Z to 256 × 256 × 128. The effect of downsampling is illustrated in Figure 6e. This process is expected to increase the computational efficiency for the steps that follow.

#### 3.1.4. Defect Generation

**Figure 7:**
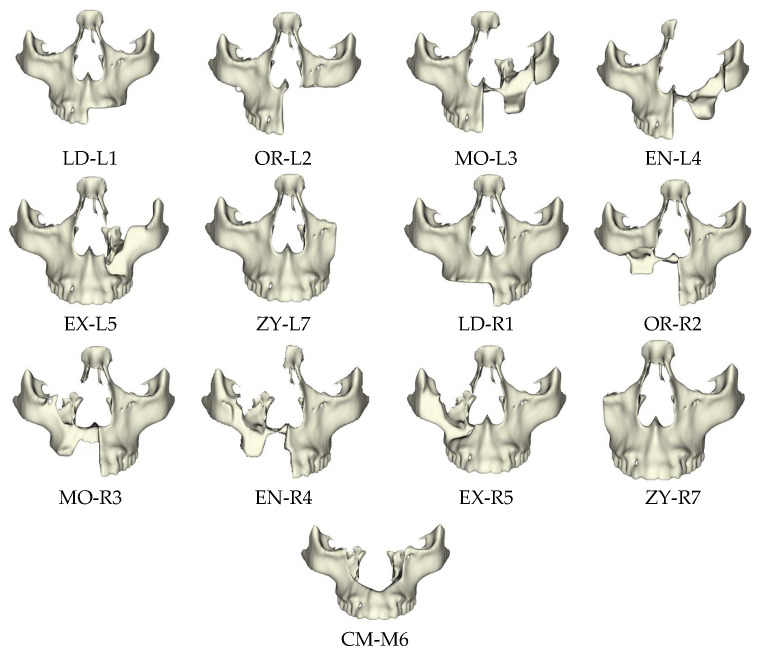
Maxillae with Defects created at the Seven Identified Regions on a Representative Maxilla.

This step marks the end of the data preparation pipeline, as shown in Figure 6f. The defects created according to the seven identified regions in Section 2.1.5 resulted in the defectied maxillae presented in Figure 7. The input and output dimensions of the maxillae remain the same, i.e., 256 × 256 × 128, while extraneous structures not required for model training are removed through CCA. Once the defects were created, the files were downsampled to 128 × 128 × 64 to be input into the model.

#### 3.1.5. Dataset Description

Table 3 provides a graphical summary of the dataset generated using the clinically acquired and preprocessed maxillary data. The 921 healthy samples were synthetically injected with the 13 defect masks described in Section 2.1.5, resulting in a total of 11,973 defect–implant data pairs (921 healthy samples × 13 defect masks).

Of the complete dataset, 20% was reserved exclusively for testing, while the remaining 80% was utilised for model development. Within this 80% development subset, 70% of the data pairs (6723 pairs) were used for training and the remaining 30% (2873 pairs) were used for validation.

The dataset was divided to ensure an approximately equal representation of all 13 defect categories across the training, validation, and test sets. Furthermore, to prevent data leakage and overlap between subsets, the dataset split was performed strictly at the patient level. All augmented variants and defect–implant pairs corresponding to a single patient were exclusively maintained within the same subset. Consequently, no patient-specific information was shared across the training, validation, and testing datasets, thereby ensuring complete dataset isolation during model evaluation.

### 3.2. AI Modelling Results

#### 3.2.1. Quantitative Evaluation of Proposed and State-of-the-Art (SOTA) Models

To benchmark the performance of the proposed MaxI-Net, several SOTA deep learning models commonly employed for volumetric reconstruction and segmentation were evaluated under identical training conditions for fair comparison. The comparative set included UNet* [31], UNETR* [32], SegNet* [33], SwinUNETR* [34], Residual UNet* [35], SCAINet* [36], and SegResNet* [37], where * denotes minor architectural modifications required for compatibility with the curated 3D CBCT dataset. These adjustments primarily involved the use of sigmoid activation in the final layer for normalised volumetric predictions. Additionally, SegNet* was adapted from 2D to 3D operations to support volumetric inputs. Table 4 summarises the architectural complexity of all evaluated models in terms of trainable parameters and required modifications. UNETR* exhibited the highest complexity with 146.40 million parameters, followed by SwinUNETR* with 62.18 million parameters. In contrast, lightweight models such as UNet Variant* and SCAINet* contained significantly fewer parameters. The proposed MaxI-Net, with 28.20 million trainable parameters, demonstrates a balanced trade-off between model complexity and reconstruction performance. For reproducibility, all models were trained using an identical configuration described in Section 2.2.2. Specifically, training was conducted for 200 epochs with an initial learning rate of 1 × 10^−4^, batch size of 4, and gradient accumulation steps of 4 to improve memory efficiency and stabilise optimisation. All models used Dice loss optimised via Adam Optimiser under identical computational settings.

Quantitative performance was assessed by comparing reconstructed implants against ground truth using seven standard evaluation metrics capturing volumetric and geometric accuracy. The Dice Similarity Coefficient (DSC) [38] and Jaccard Similarity Coefficient (JSC) [38] measure the volumetric overlap between reconstructed and true defect regions, where higher scores indicate better defect recovery. The 95th percentile Hausdorff Distance (HD95) [39] and Average Symmetric Surface Distance (ASSD) [40] quantify surface deviation between reconstructed and true implant geometries, with lower values reflecting smoother and anatomically compliant fitting to complex maxillofacial contours. Sensitivity [41] and precision [36] measure the ability to accurately recover defect volumes without introducing excessive implant mass, where higher values are preferred. Specificity [36] evaluates the ability to avoid reconstruction of healthy bone regions, indicating cleaner and clinically sensible reconstructions when high. Table 5 summarises the comparative results across all models, ordered by increasing DSC. Interpretation of the performance trends is further supported by violin plots illustrating the distribution characteristics of each metric.

Taking an overall perspective, MaxI-Net leads the SOTA architectures across most metrics. The results achieved by MaxI-Net are closer to the ideal values than those obtained by other models. MaxI-Net achieves the highest values in metrics that directly quantify volumetric similarity between the ground truth defect regions and their reconstructions, such as DSC and JSC. Specifically, MaxI-Net achieved 0.778 and 0.645 for DSC and JSC, respectively, which indicates high structural conformity between reconstructed defect volumes and the ground truth. SegResNet* follows with a DSC of 0.769, while UNet Variant* records the lowest value at 0.687. It is worth noting that the absolute DSC and JSC values remain relatively low across all models, which can be attributed to the anatomical complexity of the maxillary and zygomatic regions, making defect reconstruction inherently challenging.

In addition to volumetric reconstruction accuracy, MaxI-Net also exhibits superior boundary similarity by attaining the lowest values of the 95th percentile HD95 and ASSD, with 3.453 and 1.018, respectively. These metrics are sensitive to surface discrepancies and can return high values even when volumetric overlap is good. The minimal scores achieved by MaxI-Net therefore indicate tighter boundary alignment and better geometric precision in reconstructing defect surfaces.

**Figure 8:**
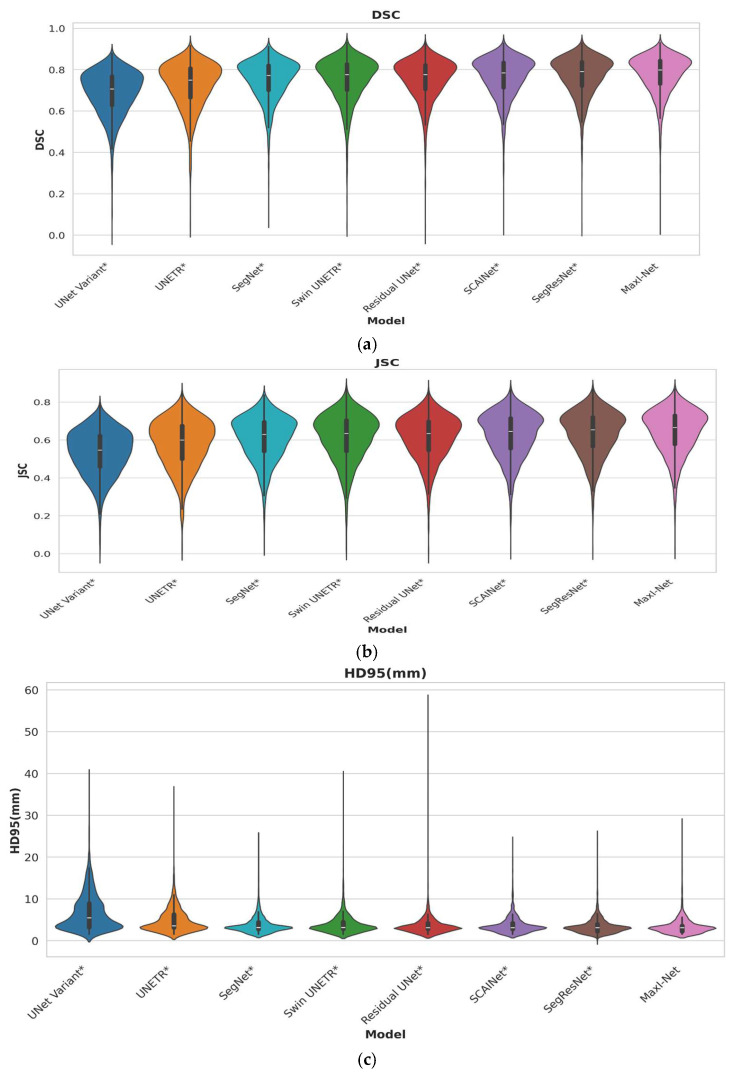

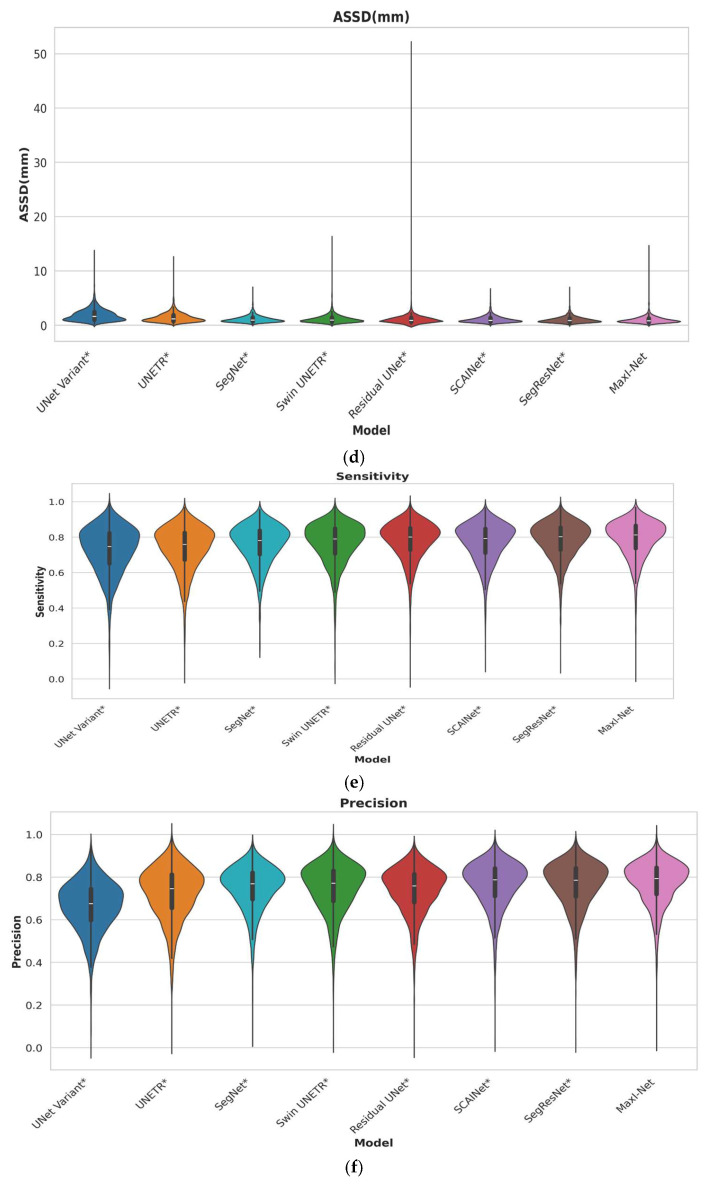

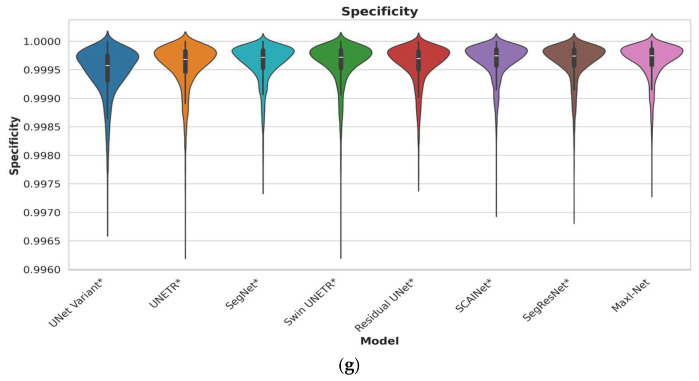
Violin Plots to Visualise the Performance of the Proposed and SOTA Models on (**a**) DSC, (**b**) JSC, (**c**) HD95, (**d**) ASSD, (**e**) Sensitivity, (**f**) Precision, and (**g**) Specificity.

MaxI-Net also leads the standard classification metrics including sensitivity, precision, and specificity, with values of 0.790, 0.774, and 0.9997, respectively. SegResNet* and SCAINet* follow, although they still trail MaxI-Net in terms of reconstruction accuracy. High sensitivity indicates that most of the true positive defect voxels in the ground truth (binary value 1 in NRRD data) are correctly predicted as defects by the model. High precision implies reduced noise generation since most voxels predicted as defects by the model were true defect voxels. Specificity is also desirable since it reflects the correct identification of background regions. However, high specificity alone can be misleading if sensitivity and precision remain low since this suggests that the model is incorrectly labelling defect voxels as background. This trend is observed across most SOTA architectures, likely due to class imbalance since background voxels outnumber defect voxels. MaxI-Net is comparatively less affected by class imbalance and achieves the highest sensitivity and precision values among all tested models. Figure 8 provides a comprehensive visualisation of the comparative performance through violin plots. The quantitative outcomes in Table 5 are also reflected in these plots, where MaxI-Net exhibits higher median values for positive metrics and lower median values for negative metrics.

As shown in Figure 8a,b, the violin plots for DSC and JSC reveal higher positioning and broader upper regions for MaxI-Net compared to SOTA architectures, indicating strong performance across a large number of test cases. For HD95 and ASSD, Figure 8c,d shows smooth distributions for MaxI-Net, while models such as UNet Variant* display slight bimodal patterns, with density shifting toward larger error values, which is undesirable. Again, MaxI-Net achieves the lowest median values for both metrics. The violin plots for sensitivity, precision, and specificity in Figure 8e–g illustrate narrow interquartile ranges for MaxI-Net, indicating consistent performance across cases. A notable trend is the small tail observed in SegResNet* and SCAINet*, indicating the absence of extreme failure cases. MaxI-Net shares this characteristic to a lesser degree while still maintaining superior median performance.

#### 3.2.2. Distribution and Variability Analysis of Reconstruction Metrics

To complement the average performance analysis, we further evaluated median, Standard Deviation (SD), minimum (Min), maximum (Max), and 95% confidence interval (CI) for DSC and HD95, as presented in Table 6 and Table 7, respectively. These additional statistical measures provide insight into the central tendency, variability, reliability, and best- and worst-case reconstruction outcomes across the testing cohort, which are important for assessing the robustness of clinically applicable maxillofacial defect reconstruction frameworks.

From Table 6, MaxI-Net achieves the highest median DSC (0.799) with one of the lowest SD values (0.094), indicating strong volumetric overlap and consistent reconstruction performance across subjects. In contrast, UNet Variant* (median = 0.707) and UNETR* (median = 0.750) exhibit substantially lower overlap, reflecting reduced reconstruction capability in anatomically complex defect scenarios.

The CI analysis further supports the reliability of MaxI-Net. Using the standard 95% confidence interval [42] computed from the sample mean and standard error, MaxI-Net achieved a DSC confidence interval of (0.775, 0.782), representing the highest lower and upper confidence bounds among all compared architectures. The narrow confidence interval width (0.007) reflects low uncertainty in the estimated mean performance across the large testing cohort of 2377 cases, while the elevated interval position indicates consistently superior volumetric reconstruction performance relative to competing architectures.

As shown in Table 7, MaxI-Net records a median HD95 of 3.145 mm, which is jointly the lowest value together with SegResNet*, indicating strong boundary conformity and reduced geometric reconstruction error. The HD95 confidence intervals reveal that SegResNet* achieves a marginally lower mean boundary error (CI: 3.390, 3.550) compared to MaxI-Net (CI: 3.480, 3.661), suggesting near-equivalent but slightly superior surface precision for SegResNet* on average.

However, SegResNet* demonstrates comparatively lower volumetric similarity performance (DSC CI: 0.765, 0.773) relative to MaxI-Net (0.775, 0.782), and its DSC confidence interval lies entirely below that of MaxI-Net with no overlap, further supporting the superior volumetric reconstruction consistency achieved by MaxI-Net. Consequently, MaxI-Net demonstrates the most favourable balance between high volumetric overlap and competitive surface boundary accuracy among all evaluated architectures.

To rigorously validate these observed differences beyond descriptive statistics, pairwise Wilcoxon signed-rank tests [43] were conducted between MaxI-Net and all competing architectures using both DSC and HD95 metrics, with rank-biserial correlation computed as the corresponding effect size measure. The Wilcoxon signed-rank test was selected over parametric alternatives due to the bounded and non-normal distribution characteristics of DSC and HD95 scores.

Table 8 presents pairwise statistical comparisons between MaxI-Net and all SOTA architectures using the Wilcoxon signed-rank test, alongside rank-biserial correlation coefficients as effect size measures [43]. For DSC, all pairwise comparisons yielded highly significant *p*-values, confirming that the volumetric reconstruction advantage achieved by MaxI-Net is statistically robust across all competing architectures and is not attributable to sampling variability. Large effect sizes were observed against UNet Variant* (r = 0.971), UNETR* (r = 0.847), and SegNet* (r = 0.690), indicating substantial practical improvements in volumetric overlap.

Against the closest competitor, SegResNet*, a statistically significant DSC improvement was observed (*p* = 4.10 × 10^−26^, r = 0.250), reflecting a small-to-medium but consistent practical advantage across the testing cohort. Similarly, statistically significant DSC improvements were also observed against SCAINet (*p* = 4.22 × 10^−64^, r = 0.400), further supporting the robustness of the proposed framework.

For HD95, statistically significant differences were observed against all evaluated architectures. The comparatively small effect size against SegResNet* (r = 0.030) indicates near-equivalent boundary accuracy between the two models, with the primary advantage of MaxI-Net lying in improved volumetric reconstruction consistency rather than substantial surface boundary superiority. Collectively, these findings validate both the statistical robustness and practical effectiveness of the proposed MaxI-Net framework for accurate 3D maxillary reconstruction.

#### 3.2.3. Computational Efficiency Assessment

To assess computational efficiency, both inference time and training time per epoch were compared across architectures. These metrics are relevant for evaluating scalability in clinical pipelines, where fast turnaround and efficient training are essential.

As shown in Table 9, MaxI-Net exhibits a balanced computational profile, achieving an inference time of 0.060 s and a training time of 9.60 min per epoch. This places MaxI-Net close to lighter architectures such as UNet Variant*, SegNet*, Residual UNet*, and SCAINet*, while substantially surpassing them in reconstruction accuracy (see Section 3.2.1 and Figure 8). Compared to transformer-based models such as SwinUNETR* and UNETR*, MaxI-Net offers a clear efficiency advantage, reducing training time by approximately 3 to 4 times (9.60 vs. up to 38 min per epoch) while avoiding their high inference latency (0.237 s). Similarly, compared with SegResNet*, which produced competitive reconstruction results but required 0.119 s inference and 19.04 min training per epoch, MaxI-Net delivers comparable or improved performance at nearly half the runtime cost. These trends suggest that MaxI-Net offers the most favourable trade-off between reconstruction accuracy and computational efficiency, outperforming lighter architectures in reconstruction quality and heavier architectures in computational demand. This balance makes MaxI-Net well suited for time-sensitive clinical workflows, where anatomical precision and deployability must be simultaneously satisfied.

### 3.3. Post-Processing Results

**Figure 9:**
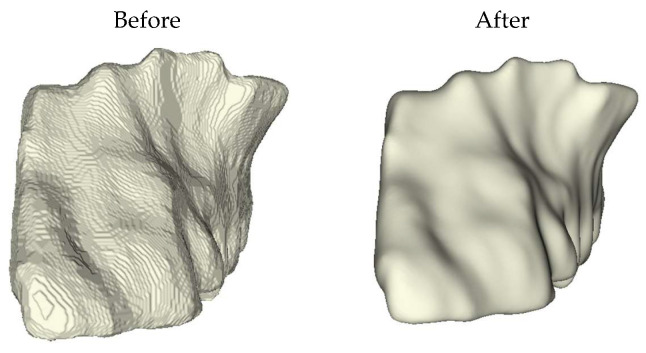
Visual Representation of the Reconstructed Defect Before and After Post-processing.

The post-processing pipeline described in Section 2.3 was applied to the reconstructed outputs, including surface smoothing, anatomical alignment, intersection removal, and gap correction. In addition, internal cavity filling of the AI-generated implant was performed as a brief manual reinforcement step prior to biomechanical evaluation. The visual comparison of the reconstructed models before and after post-processing is presented in Figure 9, demonstrating improved geometric continuity and structural completeness following post-processing.

### 3.4. Mechanical Load Testing Results

#### 3.4.1. FEM for Assembly

The FEM was conducted utilising ANSYS Workbench 2023 R1. To ensure the accurate representation of the intricate anatomical features, fine tetrahedral meshing was utilised. All constituent materials, namely the bone, the PEEK implant, and the Titanium Alloy fixture plates and pins, were modelled as linearly elastic, homogeneous, and isotropic. Their mechanical characteristics, including Young’s modulus, density, yield strength, and Poisson’s ratio, were taken from the established literature. The complex geometry of the assembly was discretised using tetrahedral elements. A mesh convergence study was subsequently performed to confirm the independence of the solution (with less than a 5% deviation). The specific material properties for all assembly components are detailed in Table 10.

#### 3.4.2. FEA for Load Testing

**Figure 10:**
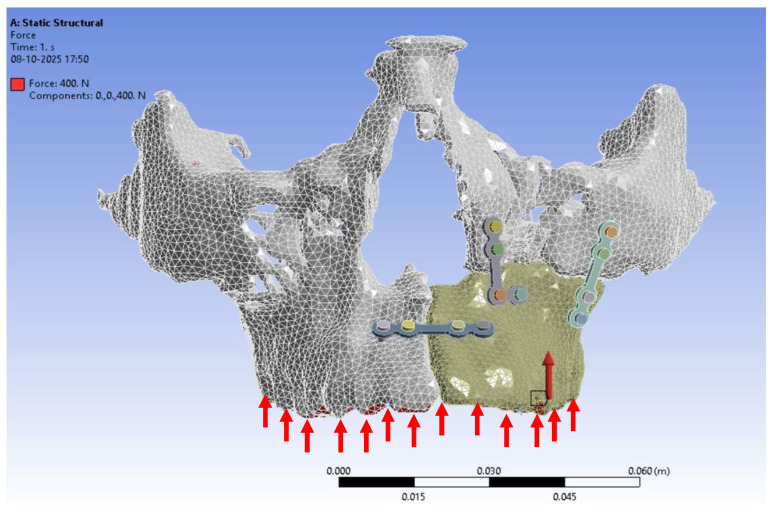
FEA for the Maxilla–Implant–Fixture Plate Assembly under Mastication Forces.

**Figure 11:**
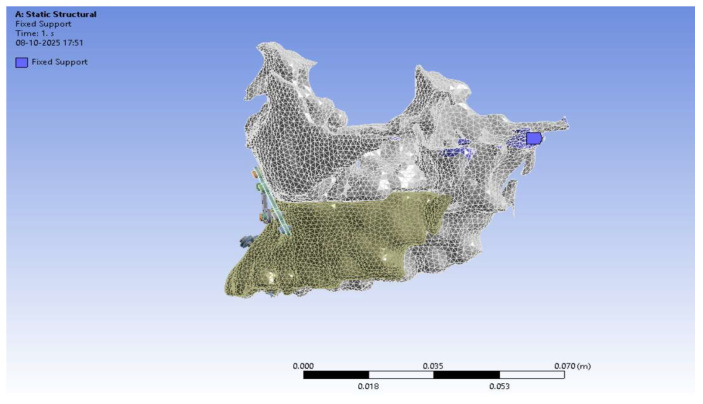
Representation of Fixed Support (Blue region) of the Maxilla.

**Figure 12:**
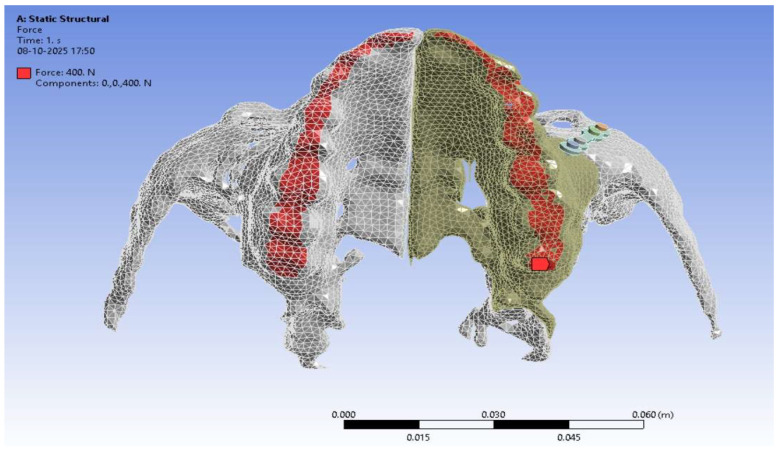
Force Representation (Red region) on the Maxilla–Implant–Fixture Assembly.

FEA served as a predictive tool to analyse the stress behaviour of the assembly under static and cyclic external loading conditions of 100 N, 250 N, and 400 N, respectively. The assembly arrangement under analysis is shown in Figure 10. Figure 11 shows the region of fixed support highlighted in blue, where the boundary condition simulates the maxilla’s rigid connection to the rest of the cranium and provides a stable base for the analysis, and Figure 12 shows the region of force application, highlighted in red. It involved applying force in an upward direction on the maxilla, as shown by the red-highlighted region. This condition mimics the mastication forces as experienced during human biting and chewing activities.

**Figure 13:**
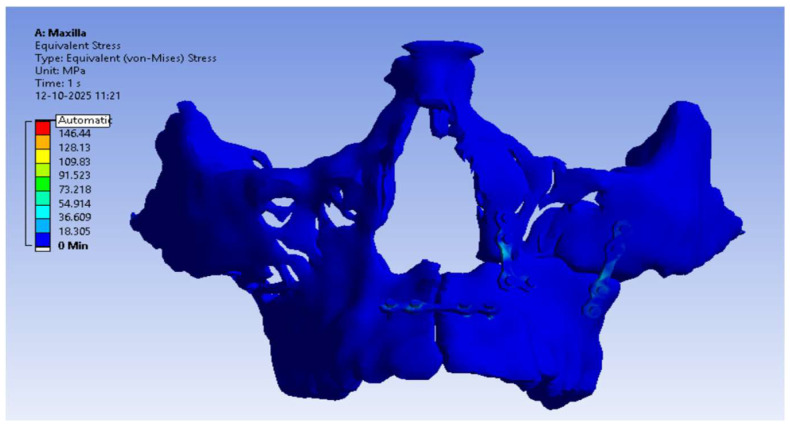
Equivalent von Mises Stress Distribution on the Maxilla–Implant–Fixture Plate Assembly with Original MaxI-Net-Generated Implant.

Static and cyclic loading forces were applied to simulate bite-force scenarios at three different magnitudes. Fatigue analysis determined the minimum number of cycles to failure and assessed long-term durability under repeated loading. For static analysis, peak von Mises stress distributions were evaluated. Under external loads of 400 N, 250 N, and 100 N, peak von Mises stresses of 658.96 MPa, 411.85 MPa, and 164.74 MPa were observed within the assemblies. As the peak stresses were concentrated in the Titanium Alloy fixture plates (Figure 13) with a yield limit of 800 MPa, the assemblies were considered safe against failure under static loading. Cyclic loading results indicated that the assemblies sustained between 4924 and 365,519 cycles before failure. Assuming an average of approximately 1400 cycles per day for the human maxillofacial structure, the corresponding lifespan ranged from 3 to 261 days, indicating poor long-term fatigue performance of this assembly configuration.

In order to improve the fatigue lifespan of the assembly, the internal structure of the defectied maxillofacial region and the generated implant was further investigated. Human maxillofacial bone naturally contains multiple internal cavities and hollow sections distributed across the anatomical structure. Since the MaxI-Net-generated implant preserved these anatomical characteristics, it was observed that several fixture pins and plates passed through unsupported cavity regions, thereby creating mechanically weak anchorage zones. Under external mastication loading, these unsupported regions increased the vulnerability of the fixation system and contributed to reduced fatigue lifespan.

To address this limitation, an internal reinforcement structure composed of the same implant material (PEEK) was introduced within the MaxI-Net-generated implant using the boundary fill tool in Autodesk Fusion 360. The reinforcement filled the internal cavities surrounding the fixation regions and provided improved anchorage support to the fixture plates and pins, thereby reducing stress concentration and improving fatigue resistance under cyclic mastication loading.

This reinforcement resulted in only a minor increase in implant mass, from 24.68 to 26.53 g. However, the biomechanical improvements observed under identical loading conditions as shown in Table 11 were substantial. The reinforced implant–fixture assembly (Case 2) demonstrated nearly seven-fold improvement in fatigue life under 400 N loading compared to the non-reinforced configuration (Case 1), while the estimated lifespan under standar3.d mastication loading improved from approximately 9 months to nearly 24+ months.

To further improve the robustness of biomechanical validation, an additional Maxilla–Implant–Fixture assembly configuration (Case 3) with modified loading/boundary characteristics was also evaluated. The obtained results demonstrated fatigue behaviour and stress trends comparable to the reinforced implant assembly (Case 2), thereby supporting the consistency of the proposed reinforcement strategy.

As per the limitations [45] of the simulation studies, an upper threshold of 1,000,000 cycles was observed for 100 N. Basquine’s law [46] as given in Equation (1) was used.

(1) $$N=A*\sigma^{b}$$

where *N* represents the number of cycles to failure, σ denotes peak von Mises stress (MPa), and *A* and *b* are material constants.

Based on the simulated fatigue values obtained for the 400 N and 250 N loading cases, the estimated fatigue life for the reinforced implant assembly (Case 2) under 100 N loading was approximately 4.7 million cycles, corresponding to nearly 9 years of functional lifespan under standard mastication forces. Similarly, for Case 3, the estimated fatigue life under 100 N loading was approximately 5.74 million cycles, corresponding to nearly 11 years of functional lifespan. The comparable performance observed across both reinforced configurations further supports the biomechanical feasibility of the proposed implant design strategy.

### 3.5. Results of Physical Validation for Geometrical Fit

**Figure 14:**
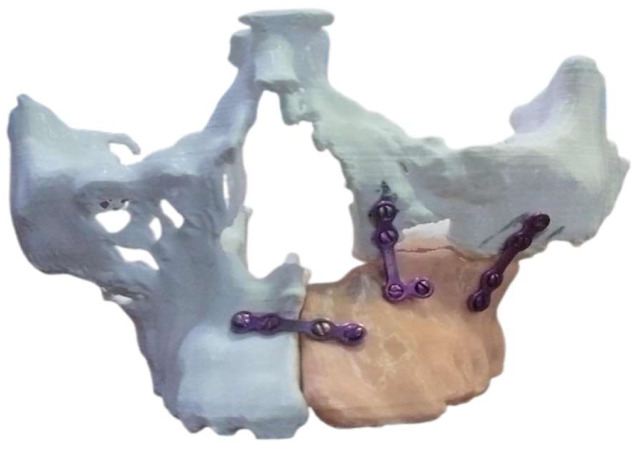
Physically Reproduced Maxilla–Implant–Fixture Plate Assembly for Purpose of the Validation for Geometrical Fit.

This section presents the physical validation of ready-to-use maxillofacial implants generated by the proposed deep learning-based reconstruction framework. A representative case was chosen for defect reconstruction from the test set of the dataset. The maxillary implant shown in Figure 14 was validated for its shape, fit, and internal–external bulging by a maxillofacial surgeon from PGIMER, Chandigarh. The implant was found to be satisfactory regarding its shape, edge gaps, and fit. Furthermore, there were no visible internal or external bulges on the implant surface.

### 3.6. Assessment and Validation Results on a Patient Case

**Figure 15:**
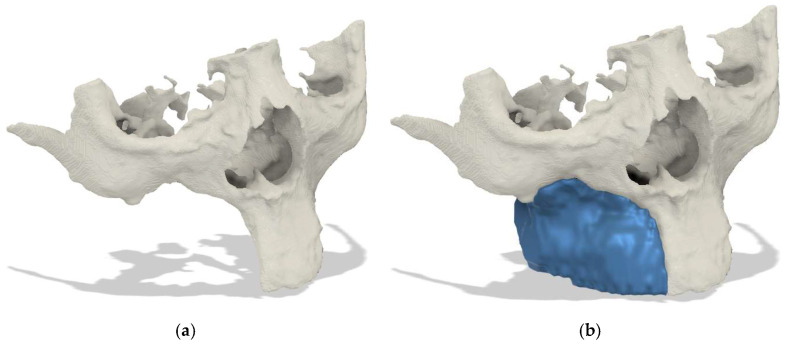
Assessment of MaxI-Net on a Real Maxillary Defect Case: (**a**) Defect-containing Maxilla and (**b**) Maxilla with the Reconstructed Defect.

The patient case presented in Figure 15 was obtained from OHSC, PGIMER. Figure 15a presents the defect-containing maxilla involving a large unilateral maxillary defect, while Figure 15b presents the reconstructed defect generated by the MaxI-Net assembled with the defectied maxilla. The generated implant demonstrated accurate anatomical conformity with the surrounding maxillary boundaries and exhibited precise fitting within the defect region. Furthermore, the reconstruction outcome was evaluated by an experienced maxillofacial surgeon and was found to be anatomically well-fitted, structurally consistent, and acceptable for implant planning.

## 4. Discussion

Maxillary and zygomatic reconstruction have traditionally relied on manual CAD modelling, requiring expert intervention and iterative refinements, resulting in time-intensive and variable workflows. Although deep learning has advanced segmentation and cranial completion, the automated generation of implant-ready midface reconstructions remains limited, particularly when biomechanical validation and manufacturability are required. This study addresses this gap through MaxI-Net, a fast and resource-efficient pipeline for the direct reconstruction of midface defects and generation of lightweight, mechanically validated implants.

Quantitative results (Table 5) show that MaxI-Net outperforms existing 3D volumetric architectures. It achieved a DSC of 0.778 and HD95 of 3.453 mm with low variability (SD = 0.094), indicating improved defect recovery and anatomical boundary preservation. Higher sensitivity and precision confirm accurate reconstruction without excessive overfilling. These improvements arise from the hybrid architecture combining dilated convolutions, residual blocks, squeeze-and-excitation modules, and CBAM attention, enabling the simultaneous capture of global structure and fine anatomical detail. Narrow violin distributions further confirm stable performance across heterogeneous defect types. From a practical standpoint, MaxI-Net demonstrated strong computational efficiency, requiring 9.6 min per epoch and generating reconstructions in 0.06 s per volume. This efficiency is critical for clinical deployment in time-sensitive and resource-constrained environments.

Statistical validation using pairwise Wilcoxon signed-rank tests confirmed that improvements are not due to sampling variability (*p* < 0.001 for all DSC comparisons). Effect sizes were strong against UNet Variant* (r = 0.971) and UNETR* (r = 0.847), and moderate against SegResNet* (r = 0.250). The 95% confidence interval for MaxI-Net DSC (0.775, 0.782) lies entirely above SegResNet* (0.765, 0.773), confirming superior volumetric consistency. For HD95, MaxI-Net achieved comparable performance to SegResNet* (median 3.145 mm), with a negligible effect size (r = 0.030), indicating that its main advantage lies in volumetric accuracy rather than surface precision. Importantly, MaxI-Net uniquely balances high volumetric overlap with competitive boundary accuracy.

Performance gains are also attributed to the large, expert-curated dataset of 921 CBCT scans expanded to 11,973 defect-healthy pairs across seven anatomical regions, capturing clinically relevant variability. The controlled defect injection strategy preserved anatomical priors while avoiding oversimplified assumptions such as mirroring, enabling generalisation to asymmetric and complex midface defects. A limitation is the reliance on synthetic defects due to the absence of paired real-world reconstruction datasets. To address this, clinical validity was evaluated on a real patient case from OHSC, PGIMER, which was judged to be anatomically acceptable by an experienced maxillofacial surgeon.

Mechanical validation via finite element analysis confirmed satisfactory performance under physiological loading. At 400 N, the reinforced implant showed peak von Mises stress of 386.13 MPa, within safe limits for titanium fixation systems. Fatigue analysis indicated survival beyond 4.7 million cycles at 100 N, corresponding to a lifespan exceeding 9 years, consistent with reported PEEK-based maxillofacial implant outcomes [47,48]. Internal cavity reinforcement, performed via a brief CAD post-processing step (<2 min, <10% mass increase), significantly improved durability without affecting workflow efficiency.

Titanium fixation plates improved stability and load transfer, consistent with established craniofacial practices [42,49,50]. The porous PEEK structure enabled favourable stress distribution and reduced concentration, aligning with prior biomechanical findings [51]. Together, fixation plates and the PEEK implant acted as a hybrid system, with titanium absorbing peak loads and protecting the surrounding bone and implant from excessive stress [52,53,54], enabling safe performance under functional loading. A limitation is that materials were modelled as homogeneous, isotropic, and linearly elastic, which may not fully capture craniofacial biomechanical complexity. Therefore, long-term predictions should be interpreted with caution.

Overall, MaxI-Net provides a largely automated pipeline for defect reconstruction, implant generation, manufacturability, and mechanical validation. Compared to conventional workflows, it reduces operator dependence, design time, and variability while improving reproducibility for complex midface reconstruction.

## 5. Conclusions and Future Scope

This work presented MaxI-Net, a deep learning pipeline that automates maxillofacial defect reconstruction and patient-specific implant generation. The framework combines a hybrid 3D model, an expert-curated and defect-augmented dataset, and a structured post-processing workflow to output implants that are readily compatible with additive manufacturing. In doing so, MaxI-Net replaces traditional CAD-based implant design workflows that are slow, operator-dependent, and difficult to scale for trauma and oncologic reconstruction. The pipeline successfully reconstructed complex maxillary and zygomatic anatomy with high accuracy while reducing implant generation time and manual effort, making it suitable for high-throughput surgical and emergency environments.

A key outcome of this study is the demonstration that AI-generated implants can satisfy biomechanical requirements relevant for clinical feasibility. FEA showed that lightweight PEEK implants with reinforced cavities and titanium fixtures can withstand physiologically meaningful mastication forces and achieve multi-year fatigue lifespans. This indicates that AI-based reconstruction can move beyond geometric shape completion toward functional implant synthesis that accounts for both anatomical fit and structural strength. These characteristics are particularly important in trauma, oncologic resection, and reconstructive surgery where rapid, patient-specific, and mechanically reliable implants are needed.

The dataset generated in this work also provides a valuable resource for future research in midface defect modelling and classification. By reducing dependency on specialised CAD operators and minimising design variability, the framework improves reproducibility and supports deployment in settings with limited design and planning resources.

Future work will prioritise the expansion of the dataset to include diverse pathological and anatomical variances, enabling continuous model fine-tuning to enhance robustness and generalisability. Additionally, the pipeline will be integrated with Augmented and Virtual Reality (AR/VR) technologies to facilitate immersive preoperative surgical planning and interactive surgical training. These technical advancements will be accompanied by the development of automated defect classification and multi-material implant designs. Finally, the framework will undergo rigorous multi-institutional validation and clinical evaluations to assess intraoperative fit, functional outcomes, and long-term implant performance.

## Figures and Tables

**Table 1:** Comparative Study of the Existing Approaches.

Year	Author	Dataset	Methodology	Merits	Demerits
2014	Iyer and Thankappan [7]	–	Review of maxillofacial reconstruction techniques and complications	Considerable clinical insights	No ideal defect classification
2018	Ji-hyeon Oh [8]	–	Review of CAD/CAM techniques for cranio-maxillofacial reconstruction	Covered manufacturing processes and implant materials	Limited to CAD/CAM
2018	Han et al. [2]	3 patients	CAD/CAM and 3D-printed PCL scaffolds	Successful anatomical reconstruction	Limited material analysis
2020	Alasseri and Alasraj [9]	6 patients	Patient-specific PEEK and titanium implants	No complications or infections	Expensive, hard-tissue focus
2020	Kong et al. [10]	2602 panoramic X-rays	EED-Net	High segmentation accuracy	No multi-class segmentation
2021	Wang et al. [11]	34 patients	VSP and 3D printing vs freehand surgery	Improved accuracy and symmetry	No soft tissue integration
2022	Lim et al. [12]	16 patients	Patient-specific titanium implants	High durability and satisfaction	High cost
2022	Choi et al. [13]	45 patients	U-Net based CBCT segmentation	Automated and efficient	Small dataset
2023	Kudva et al. [14]	4 cases	VSP and additive manufacturing	Improved rehabilitation	Complex occlusion simulation
2023	Tao et al. [15]	130 CBCT scans	VGG16 and 3D U-Net	Fast and accurate segmentation	Limited to zygomatic bone
2024	Minhas et al. [16]	123 patients	Pan2CBCT (GAN-based)	Reduced CBCT dependency	Limited clinical applicability
2024	Melerowitz et al. [6]	82 CT scans	3D U-Net segmentation	Robust and automated	Limited generalisation
2025	Matros et al. [17]	20 patients	VSP, 3D-printed plates, IDIP	Acceptable aesthetic outcomes	Limited generalisability
2025	Wu et al. [18]	30 patients	CT/MRI fusion and segmentation	Improved reliability	Small sample size

**Table 2:** Classification of Maxillofacial Defect Types and Corresponding Notations used in this Study.

Class	Defect Type	Acronym	Anatomical Description	Notation Used in This Study
I	Low Defect	LD	No orbital or nasal involvement; limited to alveolar or low palatal region	LD-L1 (left), LD-R1 (right)
II	Orbital-Involving, Eye Retained	OR	Involvement of orbital floor or rim with preservation of the eye	OR-L2 (left), OR-R2 (right)
III	Moderate Orbital Defect, Eye Retained	MO	Greater orbital bone loss than Class II, with the eye preserved	MO-L3 (left), MO-R3 (right)
IV	Orbit and Eye Removed (Enucleation)	EN	Removal of eye globe with retention of orbital contents	EN-L4 (left), EN-R4 (right)
V	Orbit and Contents Removed (Exenteration)	EX	Complete removal of orbital contents	EX-L5 (left), EX-R5 (right)
VI	Complete Maxillectomy	CM	Severe midface destruction involving both orbital and nasal structures, often crossing the midline	CM-M6 (central)
VII	Zygomatic Region Removed	ZY	Involvement of zygomatic bone and surrounding structures	ZY-L7 (left), ZY-R7 (right)

**Table 3:** Composition of the Training, Validation, and Testing Sets.

Healthy Maxilla	Maxilla After Injecting Defect	Type of Defect	Number of Training Cases	Number of Validation Cases	Number of Test Cases	Total Number of Cases
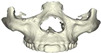	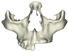	**LD-L1**	518	221	182	921
	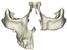	**OR-L2**	517	221	183	921
	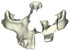	**MO-L3**	517	221	183	921
	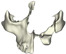	**EN-L4**	517	221	183	921
	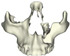	**EX-L5**	517	221	183	921
	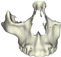	**ZY-L7**	517	221	183	921
	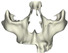	**LD-R1**	517	221	183	921
	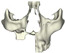	**OR-R2**	517	221	183	921
	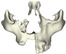	**MO-R3**	517	221	183	921
	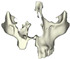	**EN-R4**	517	221	183	921
	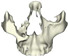	**EX-R5**	517	221	183	921
	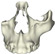	**ZY-R7**	517	221	183	921
	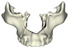	**CM-M6**	518	221	182	921
	**Combining All Defect Types**		6723	2873	2377	11,973

**Table 4:** Architectural configuration and training hyperparameters of SOTA models and MaxI-Net.

Model Name	No. of Parameters	Architectural Modification
UNet Variant*	0.01 Million	Sigmoid final layer
UNETR*	146.40 Million	Sigmoid final layer
SegNet*	21.02 Million	Sigmoid + 2D → 3D adapted
SwinUNETR*	62.18 Million	Sigmoid final layer
Residual UNet*	12.72 Million	Sigmoid final layer
SCAINet*	1.63 Million	Sigmoid final layer
SegResNet*	18.80 Million	Sigmoid final layer
MaxI-Net	28.20 Million	Proposed architecture

**Table 5:** Evaluation Results of the Proposed and SOTA Models.

Model Name	DSC	JSC	HD95 (mm)	ASSD (mm)	Sensitivity	Precision	Specificity
UNet Variant*	0.687	0.533	6.999	1.885	0.727	0.664	0.9995
UNETR*	0.725	0.579	4.835	1.432	0.737	0.725	0.9996
SegNet*	0.750	0.609	3.743	1.135	0.760	0.749	0.9996
SwinUNETR*	0.752	0.613	3.939	1.179	0.765	0.749	0.9996
Residual UNet*	0.753	0.614	3.593	1.103	0.779	0.738	0.9996
SCAINet*	0.764	0.628	3.676	1.095	0.771	0.766	**0.9997**
SegResNet*	0.769	0.635	3.470	1.030	0.783	0.764	0.9996
**MaxI-Net**	**0.778**	**0.645**	**3.453**	**1.018**	**0.790**	**0.774**	**0.9997**

Bold values represent the best performance in the metric.

**Table 6:** DSC Comparison Metrics.

Model Name	SD	Median	Min	Max	CI
UNet Variant*	0.108	0.707	0	0.878	(0.683, 0.692)
UNETR*	0.111	0.750	0.037	0.917	(0.720, 0.729)
SegNet*	0.097	0.773	0.077	0.912	(0.746, 0.754)
SwinUNETR*	0.106	0.776	0.038	**0.931**	(0.748, 0.757)
Residual UNet*	0.099	0.776	0	0.928	(0.749, 0.757)
SCAINet	0.097	0.785	0.042	0.928	(0.760, 0.768)
SegResNet*	**0.094**	0.791	0.036	0.929	(0.765, 0.773)
**MaxI-Net**	**0.094**	**0.799**	**0.043**	**0.931**	(0.775, 0.782)

**Table 7:** HD95 (mm) Comparison Metrics.

Model Name	SD	Median	Min	Max	CI
UNet Variant*	4.369	5.496	1.573	39.112	(6.524, 6.875)
UNETR*	3.011	3.578	1.573	35.628	(4.714, 4.956)
SegNet*	1.987	3.193	1.573	**25.05**	(3.664, 3.823)
SwinUNETR*	2.504	3.202	1.573	39.481	(3.838, 4.040)
Residual UNet*	2.272	3.155	1.573	57.849	(3.502, 3.684)
SCAINet	2.078	3.155	1.573	23.954	(3.592, 3.760)
SegResNet*	**1.986**	**3.145**	**0**	25.442	(3.390, 3.550)
**MaxI-Net**	2.109	**3.145**	1.573	28.327	(3.480, 3.661)

**Table 8:** Wilcoxon signed-rank *p*-values and rank-biserial effect sizes for DSC and HD95 comparisons between MaxI-Net and SOTA architectures.

	*p*-Value (*p*)		Effect Size (r)	
Model Name	DSC	HD95	DSC	HD95
UNet Variant*	<1 × 10^−300^	5.75 × 10^−305^	0.971	0.792
UNETR*	3.74 × 10^−280^	6.49 × 10^−204^	0.847	0.556
SegNet*	1.02 × 10^−186^	1.68 × 10^−43^	0.690	0.19
SwinUNETR*	1.93 × 10^−143^	6.12 × 10^−64^	0.604	0.256
Residual UNet*	1.09 × 10^−167^	8.87 × 10^−7^	0.653	0.080
SCAINet	4.22 × 10^−64^	6.47 × 10^−18^	0.400	0.216
SegResNet*	4.10 × 10^−26^	7.03 × 10^−3^	0.250	0.030

**Table 9:** Time Analysis Comparison of MaxI-Net and SOTA Architectures.

Models	Inference Times (Seconds)	Training Time per Epoch (Minutes)
UNet Variant*	0.019	3.04
UNETR*	0.166	26.56
SegNet*	0.036	5.76
Swin UNETR*	0.237	37.92
Residual UNet*	0.028	4.48
SCAINet*	0.039	6.24
SegResNet*	0.119	19.04
**MaxI-Net**	0.060	9.60

**Table 10:** Material Properties for Assembly Components [44].

Material	Elastic Modulus (MPa)	Density (kg/m^3^)	Yield Strength (MPa)	Poisson’s Ratio
Autologous Bone (Defectied Maxilla)	15,000	4430	133	0.30
PEEK (Implant)	4000	1240	100	0.44
Ti-6Al-4V (Fixture plates and pins)	110,000	4500	800	0.30

**Table 11:** Peak von Mises Stresses and Lifespan for Maxillofacial-Reinforced/Non-Reinforced Implant–Fixture Plate Assemblies when Subjected to External Loading.

External Applied Load (N)	Case 1: Maxillofacial Non-Reinforced Implant–Fixture Assembly			Case 2: Maxillofacial Reinforced Implant–Fixture Assembly			Case 3: Modified Maxillofacial Implant–Fixture Assembly		
	Peak von Mises Stress (MPa)	Minimum Number of Cycles of PEEK Implant	Estimated Minimum Lifespan (Days)	Peak von Mises Stress (MPa)	Minimum Number of Cycles of PEEK Implant	Estimated Minimum Lifespan (Days)	Peak von Mises Stress (MPa)	Minimum Number of Cycles of PEEK Implant	Estimated Minimum Lifespan (Days)
400	658.96	4924	3	386.13	29,176	20	364.112	35,630	25
250	411.85	17,202	12	241.33	162,730	116	227.57	200,610	143
100	164.74	365,519	261	96.533	1,000,000+	714+	91.028	1,000,000+	714+

## Data Availability

Data can be shared with the reader as per a reasonable request.

## Abbreviations

The following abbreviations are used in this manuscript:

AI	Artificial Intelligence
AMASSS	Automatic Multi-Anatomical Skull Structure Segmentation
AMP	Automatic Mixed Precision
AR/VR	Augmented and Virtual Reality
ASSD	Average Symmetric Surface Distance
CA	Channel Attention
CAD	Computer-Aided Design
CAM	Computer-Aided Manufacturing
CBAM	Convolutional Block Attention Modules
CBCT	Cone Beam-Computed Tomography
CCA	Connected Component Analysis
CI	95% Confidence Interval
CM	Complete Maxillectomy
CT	Computed Tomography
DICOM	Digital Imaging and Communications in Medicine
DL	Deep Learning
DSC	Dice Similarity Coefficient
EED-Net	Enhanced Encoder-Decoder Network
EN	Orbit and Eye Removed
EX	Orbit and Contents Removed
FEA	Finite Element Analysis
FEM	Finite Element Method
GAN	Generative Adversarial Network
GELU	Gaussian Error Linear Unit
HD95	95th Percentile Hausdorff Distance
JSC	Jaccard Similarity Coefficient
LD	Low Defect
MaxI-Net	Maxillofacial Implant-generation Network
MO	Moderate Orbital Defect, Eye Retained
MRI	Magnetic Resonance Imaging
MRONJ	Medication-Related Osteonecrosis of the Jaw
Max	Maximum
Min	Minimum
NRRD	Nearly Raster Raw Data
OHSC	Oral Health Sciences Centre
OR	Orbital-Involving, Eye Retained
ORN	Osteoradionecrosis
*p*	*p*-Value
PCL	Polycaprolactone
PDLLA	Poly-DL-Lactic Acid
PEEK	Polyether-ether-ketone
PGIMER	Post Graduate Institute of Medical Education and Research
r	Effect Size
SA	Spatial Attention
SD	Standard Deviation
SE	Squeeze-and-Excitation
SiLU	Sigmoid Linear Unit
SOTA	State Of The Art
STL	Standard Tessellation Language
Ti-6Al-4V	Titanium Alloy
VSP	Virtual Surgical Planning
ZY	Zygomatic Region Removed
